# Response to therapy with direct antiviral drugs in HCV-infected patients with diabetes

**DOI:** 10.1038/s41598-025-06290-5

**Published:** 2025-07-01

**Authors:** Michał Brzdęk, Dorota Zarębska-Michaluk, Piotr Rzymski, Beata Lorenc, Justyna Janocha-Litwin, Hanna Berak, Krzysztof Tomasiewicz, Marek Sitko, Włodzimierz Mazur, Ewa Janczewska, Dorota Dybowska, Jakub Klapaczyński, Anna Parfieniuk-Kowerda, Jerzy Jaroszewicz, Anna Piekarska, Robert Flisiak

**Affiliations:** 1 Collegium Medicum, Jan Kochanowski University, 25-317 Kielce, Poland; 2 Department of Gastroenterology, Medical University of Lodz, 92-213 Lodz, Poland; 3 Department of Infectious Diseases and Allergology, Jan Kochanowski University, Radiowa 7, 25-317 Kielce, Poland; 4 Department of Environmental Medicine, Poznan University of Medical Sciences, 60-806 Poznań, Poland; 5 Pomeranian Center of Infectious Diseases, Medical University, 80-214 Gdańsk, Poland; 6 Department of Infectious Diseases and Hepatology, Wrocław Medical University, 50-367 Wrocław, Poland; 7 Outpatient Clinic, Hospital for Infectious Diseases in Warsaw, 01-201 Warsaw, Poland; 8 Department of Infectious Diseases, Medical University of Lublin, 20-059 Lublin, Poland; 9 Department of Infectious and Tropical Diseases, Jagiellonian University, 31-088 Kraków, Poland; 10 Clinical Department of Infectious Diseases in Chorzów, Medical University of Silesia, 40-055 Katowice, Poland; 11 Department of Basic Medical Sciences, School of Public Health in Bytom, Medical University of Silesia, 40-055 Katowice, Poland; 12 Department of Infectious Diseases and Hepatology, Faculty of Medicine, Collegium Medicum Bydgoszcz, Nicolaus Copernicus University, 87-100 Toruń, Poland; 13 Department of Internal Medicine and Hepatology, The National Institute of Medicine of the Ministry of Interior and Administration, 02-507 Warszawa, Poland; 14 Department of Infectious Diseases and Hepatology, Medical University of Białystok, Białystok, Poland; 15 Department of Infectious Diseases and Hepatology, Medical University of Silesia, 40-635 Katowice, Poland; 16 Department of Infectious Diseases and Hepatology, Medical University of Łódź, 90-419 Łódź, Poland

**Keywords:** Diseases, Medical research

## Abstract

The clinical and metabolic interactions between hepatitis C virus (HCV) infection and diabetes mellitus (DM) are well documented. The study aimed to compare HCV-infected patients with and without DM. The analysis included 18,968 patients treated with direct-acting antivirals (DAAs) between 2015 and 2023, whose data were collected retrospectively. In the study population, 2179 patients (11.5%) were diagnosed with DM. Compared to the non-diabetic population, they were male-dominated (*p* = 0.003), had a significantly higher proportion of patients aged ≥ 50 years (*p* < 0.001), and were more burdened with comorbidities (*p* < 0.001). The most common HCV genotype was 1b with a significantly higher prevalence in the diabetic group (*p* < 0.001). Liver disease advancement was higher in diabetic patients, with 17.9% advanced fibrosis and 48% cirrhosis compared to 13.2% (*p* < 0.001) and 21.8% (*p* < 0.001) in the non-diabetic population. The effectiveness of DAA therapy in patients with DM was significantly lower compared to the population without diabetes, both in intent-to-treat analysis 93.1% vs. 94.6%, *p* = 0.015, and per-protocol analysis 96.8% vs. 97.7%, *p* = 0.0128, however, logistic regression analysis did not confirm the role of diabetes as an independent predictor of treatment failure, suggesting that in the absence of other negative prognostic factors, DM alone does not reduce the chances of cure.

## Introduction

Hepatitis C virus (HCV) infection is a leading cause of liver disease worldwide. According to the latest WHO estimates, approximately 50 million people worldwide are chronically infected with HCV, with one million new infections occurring annually^1^. In most cases, acute infection is asymptomatic, but 75–85% of infected individuals progress to chronic hepatitis C (CHC), characterized by persistent inflammation and liver fibrosis^2,3^. This progression can lead to cirrhosis in about 20% of cases, subsequently increasing the risk of liver failure and hepatocellular carcinoma (HCC)^4^. These severe complications contribute to over 242,000 deaths annually, despite the availability of safe and effective direct-acting antiviral (DAA) treatments^1^. Limited access to DAAs, particularly in low- and middle-income countries, remains a significant public health challenge^5^while development of effective vaccine to prevent the infection or its progression to CHC faces numerous difficulties^6^.

Beyond its hepatic manifestations, chronic hepatitis C is a systemic disease. An estimated 40–75% of individuals with CHC experience extrahepatic symptoms, as demonstrated by epidemiological, clinical, immunological, and pathological data^7,8^. Among the well-documented extrahepatic manifestations are autoimmune diseases and lymphoproliferative disorders, including mixed cryoglobulinemia and B-cell non-Hodgkin’s lymphoma. HCV infection is also associated with cardiovascular, neurological, and renal diseases, as well as metabolic abnormalities such as insulin resistance, type 2 diabetes mellitus (T2DM), and dyslipidemia, all of which contribute to the higher extrahepatic mortality observed in CHC patients^7,9,10^.

HCV disrupts glucose and lipid metabolism through both direct and indirect mechanisms. Viral effects on hepatocytes impair insulin signalling, while chronic inflammation and cytokine release promote systemic insulin resistance^11,12^. This metabolic dysregulation often precedes the development of T2DM in CHC patients, exacerbating liver fibrosis and creating a vicious cycle of hepatic and systemic disease progression^13^.

HCV infection has been shown to increase the risk of developing T2DM nearly fourfold compared to uninfected individuals, with the prevalence of T2DM among HCV patients ranging from 13 to 31.5%, depending on population and methodology^14–16^. Mechanisms underlying this association include HCV-induced insulin resistance, chronic inflammation, and hepatic steatosis. Conversely, pre-existing T2DM can worsen liver disease outcomes in HCV patients by accelerating fibrosis progression and reducing the efficacy of antiviral therapies^13^. This bidirectional interplay highlights the importance of addressing metabolic comorbidities in HCV management. This is especially important if one considers that T2DM accounts for about 90% of all diabetes cases^17^ and is a major cause of morbidity and mortality worldwide^18^. According to the International Diabetes Federation (IDF), 537 million adults worldwide were living with DM in 2021, a figure projected to rise to 783 million by 2045. In the same year, DM caused 1.6 million deaths globally, underscoring its public health significance^19^.

Therefore, this study aimed to investigate the clinical and metabolic interactions between HCV and T2DM by comparing two groups of HCV-infected patients treated with DAAs: those with and without diabetes. Specifically, we examined differences in clinical characteristics, severity of liver disease, treatment efficacy, and safety outcomes. In doing so, we sought not only to explore the clinical interplay between HCV and T2DM, but also to evaluate whether our findings validate previous evidence suggesting that T2DM contributes to more advanced liver disease and adverse outcomes in HCV infection. Understanding these differences provides valuable insight into optimizing care for HCV-infected patients with coexisting diabetes.

## Materials and methods

### Studied population and data collection

This analysis included data from 18,968 patients undergoing treatment for CHC with DAAs between July 1, 2015, and December 31, 2023. The data were sourced from the EpiTer-2 observational study, managed by the Polish Association of Epidemiologists and Infectiologists. The dataset comprises treatment information from 22 specialized hepatology centers across Poland, reflecting real-world therapeutic outcomes.

The selection of treatment regimens was guided by the discretion of individual physicians, following the clinical recommendations of the Polish Group of Experts for HCV and the National Health Fund in Poland^20–22^. Patient data were collected retrospectively and included a wide range of variables: demographic information (age, sex, body mass index), comorbidities, liver disease severity, HCV genotype and viral load, co-infections with human immunodeficiency virus (HIV) and hepatitis B virus (HBV), previous HCV treatments and current regimen used, as well as clinical parameters such as the presence of HCC, alanine transaminase (ALT) activity, albumin, hemoglobin, creatinine concentrations, and platelet counts.

The study was carried out with approval from the Bioethics Committee of Jan Kochanowski University in Kielce (Resolution No. 57/2024, dated July 25, 2024). All patients provided informed consent to participate in the therapeutic program in accordance with National Health Fund regulations. All methods were performed in accordance with the relevant guidelines and regulations.

### Diagnosis of diabetes mellitus

The diagnosis of DM, as well as its type, was established based on a thorough medical history and a comprehensive review of the patient’s medical records.

### Assessment of the liver disease severity

The severity of liver disease was assessed using non-invasive fibrosis evaluation techniques. These included transient elastography, performed with the FibroScan device (Echosens, France), and shear-wave elastography using the Aixplorer system (SuperSonic Imagine, Aix-en-Provence, France). Fibrosis staging was determined according to the METAVIR scoring system, which is in line with the European Association for the Study of the Liver (EASL) guidelines. A liver stiffness measurement threshold of 13 kPa was used to identify patients likely to have cirrhosis (F4)^23^.

These patients were further evaluated using the Child-Pugh (CP) scoring system to assess liver function at the start of antiviral treatment. Patients classified with a CP score of B or C were considered to have decompensated cirrhosis. Information was also collected on past decompensation of liver function and the presence of esophageal varices.

### Antiviral treatment

Patients were treated with genotype-specific or pangenotypic DAA options. The genotype-specific regimens included combinations such as: asunaprevir (ASV) + daclatasvir (DCV), ombitasvir/paritaprevir/ritonavir ± dasabuvir with or without ribavirin (OBV/PTV/r ± DSV ± RBV), ledipasvir (LDV) and sofosbuvir (SOF) ± RBV, grazoprevir (GZR) and elbasvir (EBR) ± RBV, and SOF with simeprevir (SMV) ± RBV. The pangenotypic options consisted of the combination of SOF and RBV, SOF with DCV ± RBV, SOF and velpatasvir (VEL), SOF/VEL with voxilaprevir (VOX), glecaprevir (GLE) and pibrentasvir (PIB), and GLE/PIB + SOF and RBV.

### Evaluation of treatment effectiveness

The effectiveness of antiviral therapy was assessed by measure HCV RNA in serum 12 weeks after the end of treatment. Undetectable HCV RNA at this time point indicated treatment success and achievement of a sustained virologic response (SVR12). Patients who had a detectable viral load were classified as virologic failures, while individuals who did not undergo testing due to loss to follow-up were identified as nonvirologic failures. HCV RNA was assessed by reverse transcriptase-polymerase chain reaction with a lower limit of detection not higher than 15 IU/mL following EASL recommendations^23^.

### Evaluation of treatment safety

Safety outcomes were monitored throughout the treatment period and for 12 weeks after its completion. Data were collected on any modifications or discontinuation of the treatment regimen, occurrences of adverse events (AEs), serious adverse events (SAEs), and any fatalities, with an assessment of their potential relationship to antiviral therapy. Special attention was given to AEs linked to liver function, particularly in patients with cirrhosis. This included monitoring for complications such as gastrointestinal bleeding, ascites, and hepatic encephalopathy.

### Statistical analyses

Differences in continuous variables were evaluated using the U-Mann-Whitney test. To compare event frequencies between groups with and without hypertension, χ² Pearson’s test was employed, or Fisher’s exact test was used when the number of observations was less than 5.

The entire study population was included in the intention-to-treat (ITT) analysis, while the per-protocol (PP) analysis focused on patients who had an HCV RNA assessment 12 weeks after completing treatment. Based on univariate analyses that examined factors associated with achieving or not achieving a SVR12, logistic multiple regression models were applied to estimate the odds of treatment failure both in the overall study population and specifically in patients with DM. A p-value of less than 0.05 was considered statistically significant for all analyses. Statistical analyses were conducted using Statistica v. 13 (StatSoft, USA) and MedCalc v. 15.8 (MedCalc Software Ltd, Belgium).

## Results

### General characteristics

A total of 18,968 HCV-infected patients were analyzed, of which 2179 (11.5%) had a diagnosis of DM. The diabetic group had a higher proportion of patients aged ≥ 50 years (83.0% vs. 48.6%, *p* < 0.001) and was dominated by men (53.2%), who accounted for 49.8% in the group without diabetes (*p* = 0.003) (Table 1).

**Table 1:** The comparison of baseline characteristics of patients with and without diabetes.

Parameter	All patients (*n* = 18968)	Diabetic group (*n* = 2179)	Non-diabetic group (*n* = 16789)	*p*-value
Age, mean (SD)	51.2 (14.6)	61.1 (12.0)	49.9 (14.5)	< 0.001 (U = 10174088)
Age ≥ 50, % (n)	52.6 (9969)	83.0 (1809)	48.6 (8180)	< 0.001 (*χ*^2^ = 916.2)
Men/Women, % (n)	50.2/49.8 (9520/9448)	53.2/46.8 (1159/1020)	49.8/50.2 (8361/8428)	0.003 (*χ*^2=^8.9)
BMI, mean (SD)	26.2 (4.5)	28.5 (5.2)	25.9 (4.3)	< 0.001 (U = 12106021)
Comorbidities				
Any comorbidities (exluding diabetes), % (n)	63.7 (12089)	85.4 (1861)	60.9 (10228)	< 0.001 (*χ*^2^ = 500.2638)
Obesity, % (n)	17.5 (3258)	32.7 (692)	15.6 (2566)	< 0.001 (*χ*^2=^382.4)
Arterial hypertension, % (n)	31.5 (5976)	67.6 (1473)	26.8 (4503)	< 0.001 (*χ*^2^ = 1486.2)
Autoimmune diseases, % (n)	2.0 (386)	2.1 (46)	2.0 (340)	0.789 (*χ*^2^ = 12.2)
Non-HCC tumors, % (n)	2.1 (400)	2.8 (61)	2.0 (339)	0.017 (*χ*^2^ = 5.7)
Renal disease, % (n)	3.6 (689)	9.1 (199)	2.9 (490)	< 0.001 (*χ*^2^ = 212.8)
ALT (IU/L), median (IQR)	81.4 (73.0)	89.2 (74.9)	80.4 (72.7)	< 0.001 (U = 16424357)
Albumin (g/dL), median (IQR)	4.1 (0.5)	3.9 (0.5)	4.1 (0.5)	*p* < 0.001 (U = 13667247)
Bilirubin (mg/dL), median (IQR)	0.8 (0.8)	0.9 (0.9)	0.8 (0.8)	*p* < 0.001 (U = 16362913)
Hemoglobin (g/dL), median (IQR)	14.4 (1.7)	14.0 (1.8)	14.4 (1.7)	*p* < 0.001 (U = 15572149)
Platelets (*1000/µL), median (IQR)	197.4 (78.6)	169.0 (77.5)	201.0 (78.0)	*p* < 0.001 (U = 13642364)
Creatinine (mg/dL), median (IQR)	0.9 (0.7)	1.0 (0.7)	0.9 (0.6)	*p* < 0.001 (U = 16049900)
HCV RNA, × 10^6^ IU/mL, median (IQR)	2.39 (7.14)	2.89 (6.56)	2.33 (7.20)	*p* < 0.001 (U = 16661058)

BMI, body mass index; ALT, alanine transaminase; IQR, interquartile range; HCV, hepatitis C virus; HCC, hepatocellular carcinoma; RNA, ribonucleic acid; SD, standard deviation.

Patients with DM were significantly more often burdened with comorbidities compared to the non-diabetic population (85.4% vs. 60.9%, *p* < 0.001), with hypertension being the most common in both groups (67.6% vs. 26.8%, *p* < 0.001). Obesity, defined as BMI ≥ 30 kg/m3, was also significantly more common in the diabetic group (32.7% vs. 15.6%, *p* < 0.001).

Among laboratory tests, patients with diabetes showed significantly higher ALT activity, bilirubin, creatinine, and baseline HCV RNA levels, while albumin, haemoglobin, and platelet counts were significantly lower in them compared to the group without DM (*p* < 0.001).

The most common HCV genotype in the population was GT1b, found in 73.8% of patients, with a significantly higher prevalence in the diabetic group (80.5% vs. 72.9%, *p* < 0.001). Infections with GT3 and GT4 were significantly less frequently reported in the DM population as compared to the non-diabetic group, 11% vs. 12.5%, *p* < 0.001 and 3.1% vs. 5.1%, *p* < 0.001, respectively. Liver fibrosis (F) was more advanced in patients with diabetes, with 17.9% diagnosed with F3 and 48% with F4 compared respectively to 13.2% (*p* < 0.001) and 21.8% (*p* < 0.001) in the non-diabetic population. We also documented a significantly higher percentage of patients with a history of decompensated cirrhosis in the form of ascites (*p* < 0.001) and encephalopathy (*p* < 0.001), the presence of esophageal varices (*p* < 0.001) and with decompensated cirrhosis at the start of therapy in the diabetes group compared to patients without diabetes (Table 2).

HCC was diagnosed significantly more frequently among patients with DM, 3.7% vs. 1.3% in those without DM, *p* < 0.001. HIV co-infection was significantly less common in diabetics (1.8% vs. 6.7%, *p* < 0.001), while HBV co-infection rates were similar in both groups.

**Fig. 1:**
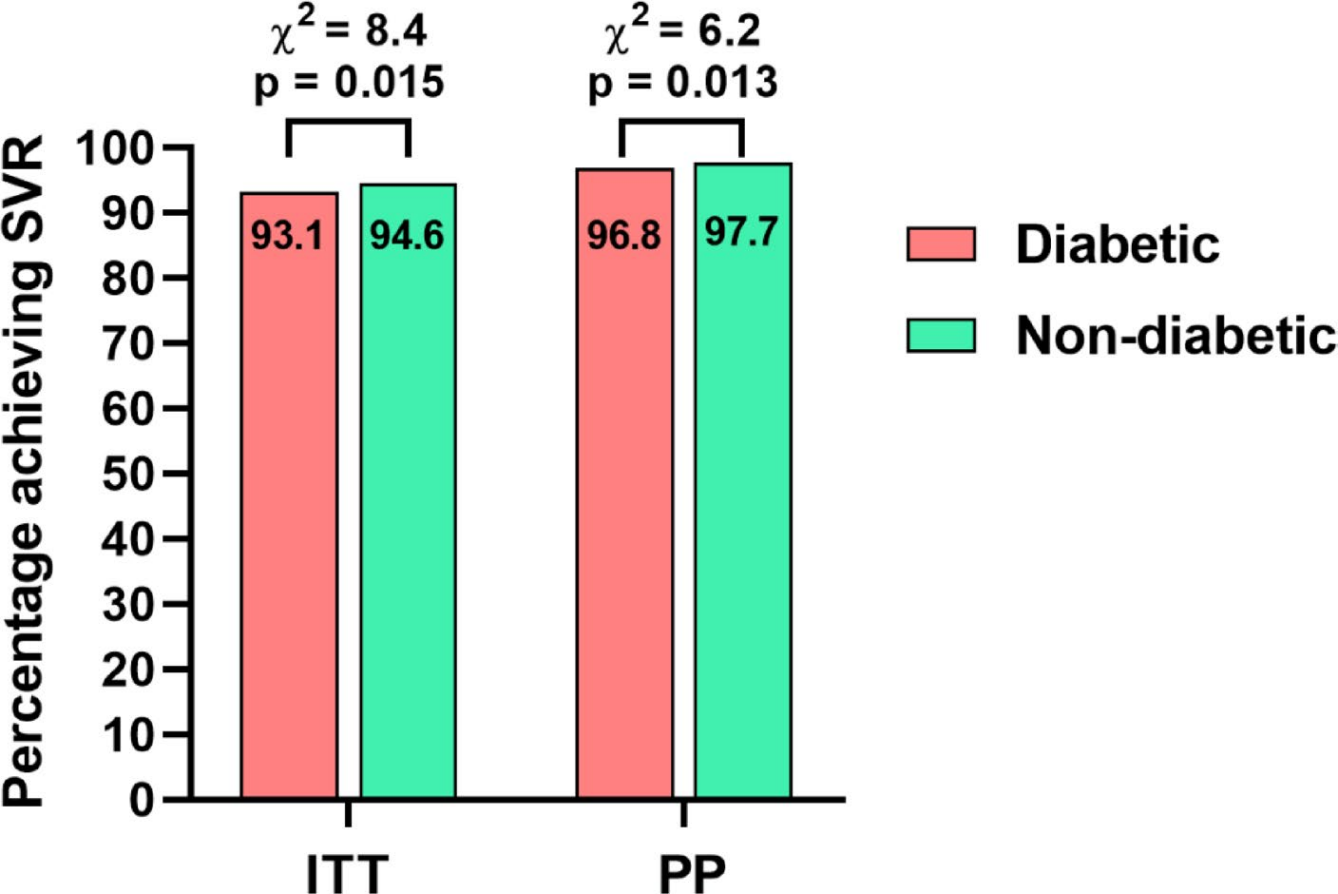
SVR12 rates in patients with and without diabetes. Abbreviations: SVR12, sustained virologic response; HCV, Hepatitis C virus; GT, genotype; F,fibrosis.

**Fig. 2:**
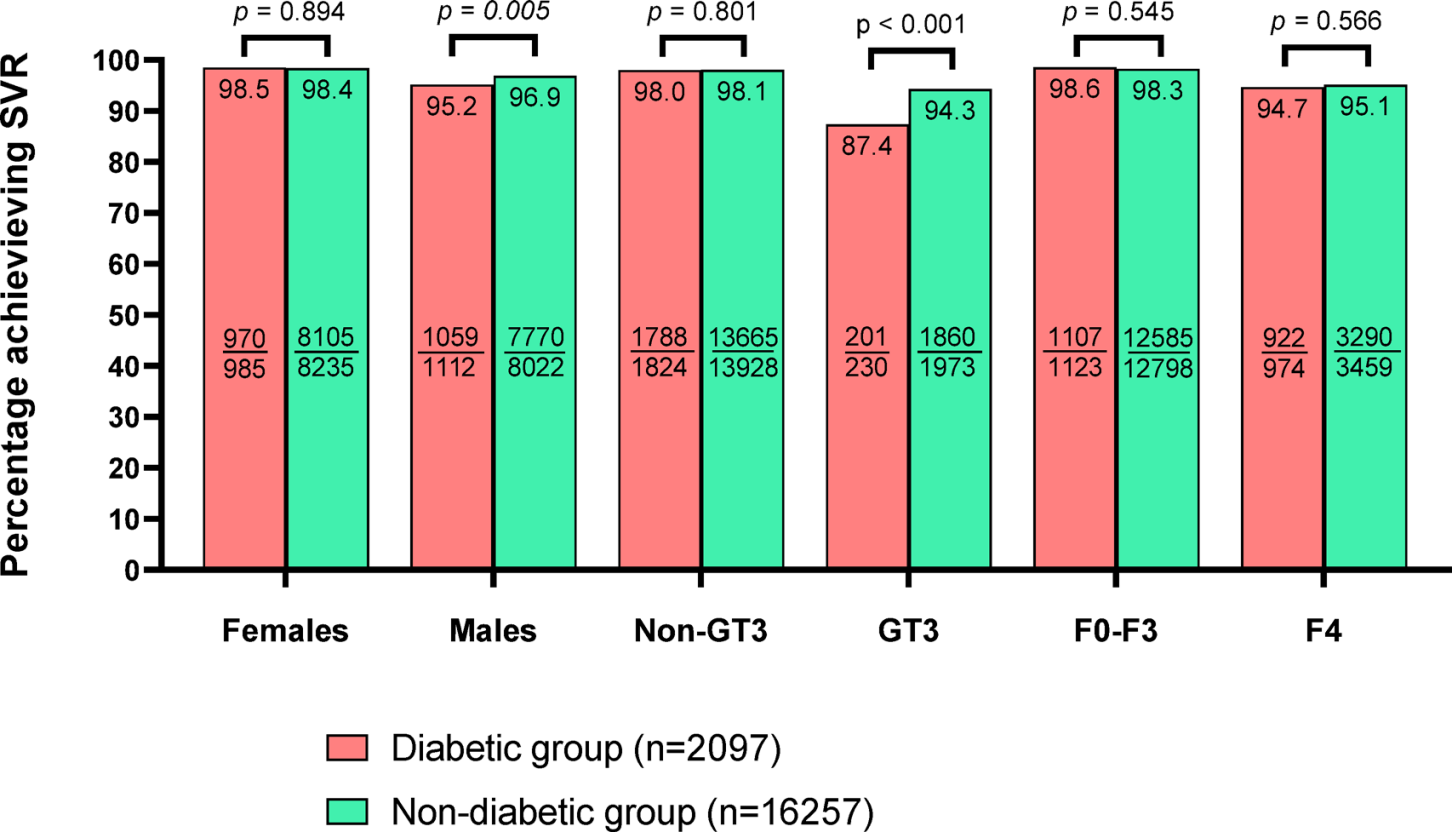
Comparison of SVR12 rates in a population of patients with and without diabetes according to gender, HCV genotype and fibrosis (per protocol analysis). Abbreviations: SVR12, sustained virologic response; HCV, Hepatitis C virus; GT, genotype; F, fibrosis.

**Fig. 3:**
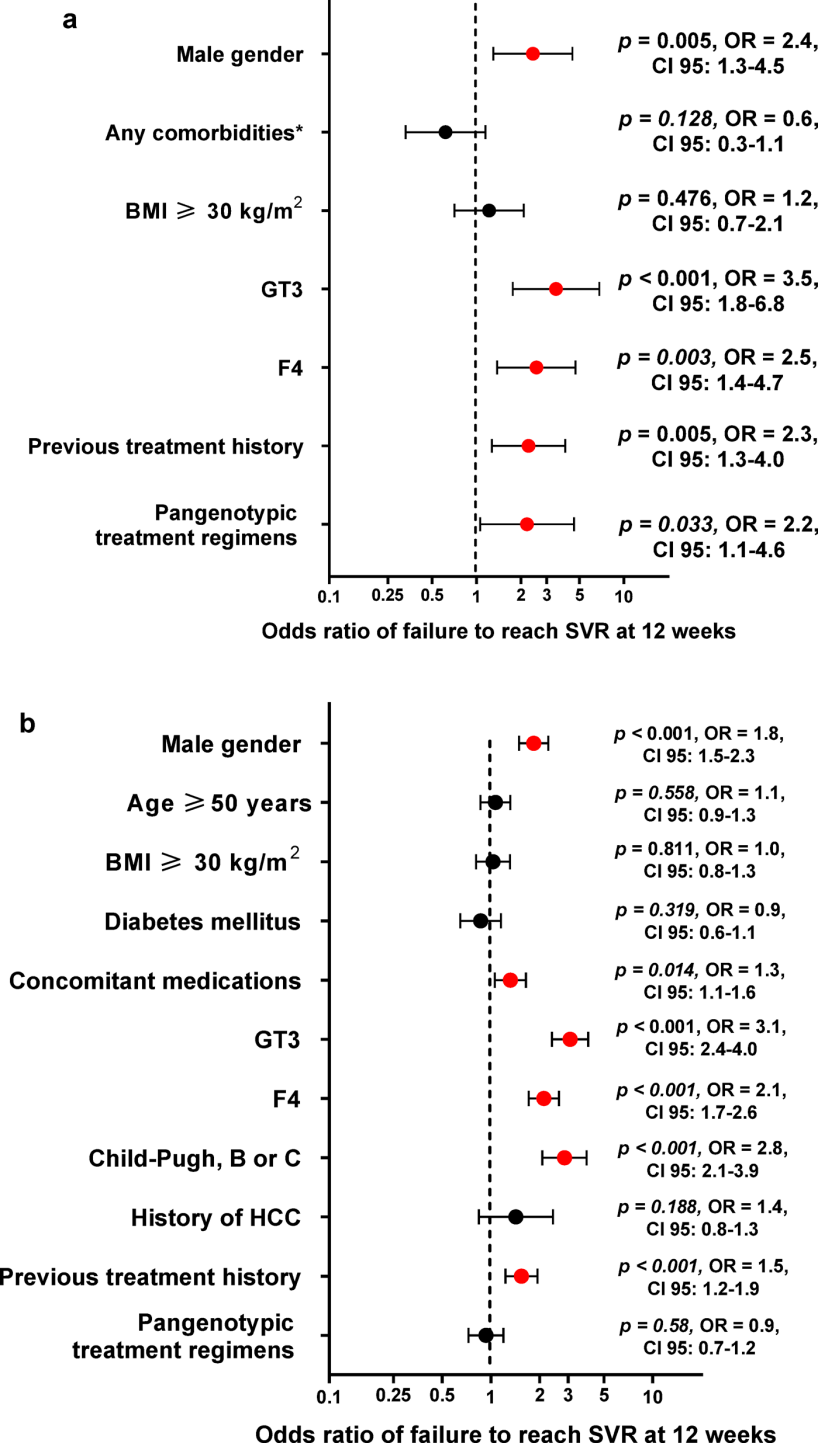
Factors associated with virologic failure in the logistic regression model in diabetic group (**a**) and in all HCV-infected patients (**b**). *excluding diabetes mellitus. Abbreviations: BMI, body mass index; GT, genotype; F, fibrosis; HCC, hepatocellular carcinoma; HCV, Hepatitis C virus; SVR12, sustained virologic response.

### Treatment characteristics

The majority of patients across all groups were treatment-naive (80.8%), although a significantly smaller proportion of diabetic patients were treatment-naive compared to non-diabetic patients (73.1% vs. 81.8%, *p* < 0.001) (Table 3).

Genotype-specific regimens were more commonly used in the DM population, 59.9% vs. 51.4% in patients without DM, *p* < 0.001. Moreover, diabetic patients were more frequently treated with RBV-containing regimens. Treatment regimens significantly more frequently used in diabetic patients included SOF/LDV ± RBV, SOF + RBV, and SOF/VEL ± RBV, while non-diabetic patients were significantly more often treated with GLE/PIB.

### Treatment effectiveness

The effectiveness of therapy in patients with DM in the ITT analysis was significantly lower compared to the non-diabetic population, 93.1% vs. 94.6%, *p* = 0.015, also after excluding LTFU patients, the difference in virological response to the detriment of diabetic patients was statistically significant 96.8% vs. 97.7%, *p* = 0.0128 (Fig. 1).

A detailed analysis comparing the effectiveness of DAA therapy calculated in a per-protocol analysis in a population of patients with and without diabetes according to gender, HCV genotype, and fibrosis showed significantly lower response rates in males and patients infected with GT3, while those with liver cirrhosis achieved comparable SVR12 regardless of the presence of DM (Fig. 2).

Among virologic non-responders, a significantly higher proportion of males, patients with a BMI consistent with obesity, elderly patients, GT3-infected individuals, treatment-experienced patients, people with cirrhosis, esophageal varices, decompensated liver function at the start of the study, HIV co-infection and those treated with pangenotypic regimens were observed compared to patients achieving SVR12. Conversely, a lower proportion of non-responders had comorbidities and GT1 infection (Table 4).

Logistic regression analyses revealed that virologic failure in patients with diabetes mellitus was independently associated with male sex [odds ratio (OR) = 2.4], presence of cirrhosis (OR = 2.5), GT3 (OR = 3.5), previous treatment history (OR = 2.3), and pangenotypic treatment regimens (OR = 2.2). Notably, no association was observed with the presence of comorbidities other than diabetes or with a BMI of ≥ 30 kg/m^2^ (Fig. 3a).

Further logistic regression analyses examining virologic failure in the entire cohort of HCV-infected patients revealed significant associations with male gender (OR = 1.8), concomitant medications (OR = 1.3), GT3 (OR = 3.1), cirrhosis (OR = 2.1), Child-Pugh B or C classification (OR = 2.8), and previous treatment history (OR = 1.5) (Fig. 3b).

**Table 2:** The comparison of characteristics of HCV infection and liver disease in patients with and without diabetes.

Parameter	All patients (*n* = 18968)	Diabetic group (*n* = 2179)	Non-diabetic group (*n* = 16789)	*p*-value
GT1a, % (n)	4.5 (860)	1.1 (24)	5.0 (836)	< 0.001 (*χ*^2^ = 67.0)
GT1b, % (n)	73.8 (13988)	80.5 (1755)	72.9 (12233)	< 0.001 (*χ*^2^ = 58.8)
GT1, % (n)	2.0 (380)	2.2 (47)	2.0 (333)	0.586 (*χ*^2^ = 0.29)
GT2, % (n)	0.28 (53)	0.1 (2)	0.3 (51)	0.084
GT3, % (n)	12.4 (2345)	11.0 (24)	12.5 (2105)	< 0.001 (*χ*^2^ = 253.1)
GT4, % (n)	4.8 (917)	3.1 (67)	5.1 (850)	< 0.001 (*χ*^2^ = 16.6)
GT5, % (n)	0.01 (1)	0 (0.0)	1 (0.01)	1.0
GT6, % (n)	0.03 (6)	0 (0.0)	6 (0.04	1.0
Unknown GT, % (n)	2.19 (416)	2.0 (44)	2.2 (372)	0.555 (*χ*^2^ = 0.34)
History of encephalopathy, n (%)	0.8 (145)	1.8 (39)	0.6 (106)	< 0.001 (*χ*^2^ = 34.1)
History of ascites, n (%)	2.8 (533)	6.0 (130)	2.4 (403)	< 0.001 (*χ*^2^ = 89.8)
Documented esophageal varices, n (%)	7.0 (1334)	14.9 (325)	6.0 (1009)	< 0.001 (*χ*^2^ = 238.8)
Liver fibrosis				
F0, % (n)	2.8 (523)	0.9 (20)	3.0 (503)	< 0.001 (*χ*^2^ = 31.1)
F1, % (n)	39.7 (7388)	18.5 (393)	42.4 (6995)	< 0.001 (*χ*^2^ = 452.8)
F2, % (n)	19.0 (3547)	14.7 (313)	19.6 (3234)	< 0.001 (*χ*^2^ = 30.4)
F3, % (n)	13.7 (2554)	17.9 (379)	13.2 (2175)	< 0.001 (*χ*^2^ = 32.6)
F4, % (n)	24.8 (4620)	48.0 (1018)	21.8 (3602)	< 0.001 (*χ*^2^ = 668.2)
CP B, % (n)	3.2 (601)	6.5 (138)	2.8 (463)	< 0.001 (*χ*^2^ = 80.4)
CP C, % (n)	0.2 (36)	0.4 (9)	0.2 (27)	< 0.001 (*χ*^2^ = 73.3)
HCC history, % (n)	1.6 (298)	3.7 (81)	1.3 (217)	< 0.001 (*χ*^2^ = 73.4)
HIV co-infection, % (n)	6.1 (1136)	1.8 (38)	6.7 (1098)	< 0.001 (*χ*^2^ = 78.0)
HBV co-infection, % (n)	14.3 (2658)	15.4 (327)	14.2 (2331)	0.118 (*χ*^2^ = 2.5)

CP, Child-Pugh; GT, genotype; HCC, hepatocellular carcinoma; HIV, human immunodeficiency virus; HBV, hepatitis B virus; F, fibrosis.

**Table 3:** Treatment characteristics in chronic hepatitis C patients with and without diabetes.

Parameter	All patients (*n* = 18968)	Diabetic group (*n* = 2179)	Non-diabetic group (*n* = 16789)	*p*-value
History of antiviral treatment				
Treatment-naive, % (n)	80.8 (15328)	73.1 (1593)	81.8 (13735)	< 0.001 (*χ*^2^ = 94.2)
Non-responder to IFN-based regimens, % (n)	17.0 (3232)	24.2 (527)	16.1 (2705)	< 0.001 (*χ*^2^ = 88.9)
Non-responder to IFN-free regimens, % (n)	2.2 (408)	2.7 (59)	2.1 (349)	0.057 (*χ*^2^ = 3.6)
Current treatment regimen				
Current-RBV-containing regimens, % (n)	14.0 (2665)	22.8 (497)	12.9 (2168)	< 0.001 (*χ*^2^ = 156.4)
Genotype- specific treatment regimens, % (n)	52.0 (9871)	59.9 (1305)	51.4 (8633)	< 0.001 (*χ*^2^ = 22.4)
ASV + DCV, % (n)	0.7 (135)	1.0 (21)	0.7 (114)	0.137 (*χ*^2^ = 2.2)
SOF/LDV ± RBV, % (n)	16.1 (3061)	19.9 (435)	15.6 (2626)	< 0.001 (*χ*^2^ = 26.6)
OBV/PTV/r ± DSV ± RBV, % (n)	21.4 (4064)	22.2 (483)	21.3 (3581)	0.370 (*χ*^2^ = 0.8)
GZR/EBR ± RBV, % (n)	13.7 (2601)	13.6 (297)	13.7 (2304)	0.905 (*χ*^2^ = 0.01)
SOF + SMV ± RBV, % (n)	0.1 (10)	0.1 (2)	0.1 (8)	0.322
Pangenotypic regimens, % (n)	48.0 (9097)	40.1 (874)	48.6 (8156)	< 0.001 (*χ*^2^ = 22.4)
SOF + RBV, % (n)	1.8 (344)	2.7 (59)	1.7 (285)	0.001 (*χ*^2^ = 11.0)
SOF + DCV ± RBV, % (n)	0.2 (46)	0.4 (8)	0.2 (38)	0.209 (*χ*^2^ = 1.6)
GLE/PIB, % (n)	26.0 (4934)	16.9 (369)	27.2 (4565)	< 0.001 (*χ*^2^ = 105.4)
GLE/PIB + SOF + RBV, % (n)	0.04 (7)	0	0.04 (7)	1
SOF/VEL ± RBV, % (n)	19.4 (3677)	22.5 (490)	19.0 (3187)	< 0.001 (*χ*^2^ = 15.2)
SOF/VEL/VOX, % (n)	0.5 (89)	0.7 (15)	0.4 (74)	0.111 (*χ*^2^ = 2.5)

CHC, chronic hepatitis C; IFN, interferon; RBV, ribavirin; ASV, asunaprevir; DCV, daclatasvir; LDV, ledipasvir; SOF, sofosbuvir; OBV, ombitasvir; PTV, paritaprevir; r, ritonavir; DSV, dasabuvir; GZR, grazoprevir; EB, elbasvir; SMV, simeprevir; GLE, glecaprevir; PIB, pibrentasvir; VEL, velpatasvir; VOX, voxilaprevir.

**Table 4:** The comparison of virological responders and non-responders to DAA therapy in diabetic group.

Parameter	Responders, *n* = 2097	Nonresponders, *n* = 68	*P*-value
Gender, females/males, % (n)	47.8/52.2 (970/1059)	22.1/ 77.9 (15/53)	< 0.001 (*χ*^2^ = 17.5)
Age (yr), mean (SD)	61.1 (12.0)	57.6 (10.2)	0.020 U= 56158,0
Age ≥ 50, % (n)	82.8 (1680)	79.4 (54)	0.468 (*χ*^2^ = 0.5)
BMI, mean (SD)	28.5 (5.2)	5.3 (29.9)	0.029 U = 55293,0
Current treatment regimen, % (n)			
Genotype-specific treatment regimens	57.8 (1173)	29.4 (20)	< 0.001 (*χ*^2^ = 21.6)
ASV + DCV	1.0 (20)	0	1.0
LDV/SOF ± RBV	20.1 407)	13.2 (9)	0.165 (*χ*^2^ = 1.9)
OBV/PTV/r ± DSV ± RBV	22.9 (464)	11.8 (8)	0.031 (*χ*^2^ = 4.6)
GZR/EBR ± RBV	13.8 (280)	4.4 (3)	0.027
SOF + SMV ± RBV	0.1 (2)	0	1.0
Pangenotypic regimens	42.2 (856)	70.6 (48)	< 0.001 (*χ*^2^ = 21.6)
GLE/PIB	17.4 (353)	10.3 (7)	0.126 (*χ*^2^ = 2.3)
SOF/VEL ± RBV	13.8 (441)	38.2 (26)	0.001 (*χ*^2^ = 10.3)
SOF/VEL/VOX	0.7 (14)	0	1.0
SOF + RBV	2.0 (40)	22.1 (15)	< 0.001 (*χ*^2^ = 103.9)
SOF + DCV ± RBV	0.4 (8)	0	1.0
Comorbidities, % (n)			
Any (exluding diabetes)	85.7 (1739)	72.1 (49)	0.02 (*χ*^2^ = 5.4)
Hypertension	68.4 (1388)	47.1 (32)	0.001 (*χ*^2^ = 10.7)
Obesity	31.8 (645)	39.7 (27)	0.116 (*χ*^2^ = 2.5)
Autoimmune diseases	2.0 (40)	4.4 (3)	0.15
Renal disease	9.0 (182)	7.4 (5)	1.0
Non-HCC tumors	2.5 (51)	4.4 (3)	0.239
GT, % (n)			
1a, % (n)	1.2 (24)	0	1.0
1b, % (n)	81.6 (1656)	45.6 (31)	< 0.001 (*χ*^2^ = 42.7)
1, % (n)	2.2 (44)	4.4 (3)	0.181
2, % (n)	< 0.1 (1)	0	1.0
3, % (n)	9.9 (201)	42.7 (29)	< 0.001 (*χ*^2^ = 75.6)
4, % (n)	3.1 (63)	2.9 (2)	1.0
Unknown, % (n)	2.0 (40)	4.4 (3)	0.15
History of previous therapy, % (n)			
Treatment-naïve	73.4 (1489)	55.9 (38)	0.001 (*χ*^2^ = 10.2)
Treatment-experienced	26.6 (540)	44.1 (30)	0.001 (*χ*^2^ = 10.2)
History of hepatic decompensation, % (n)			
Encephalopathy and/or ascites	6.0 (122)	11.8 (8)	0.058 (*χ*^2^ = 3.6)
Ascites	5.3 (108)	10.3 (7)	0.076 (*χ*^2^ = 3.1)
Encephalopathy	1.6 (33)	2.9 (2)	0.301
Documented esophageal varices, % (n)	13.8 (281)	35.3 (24)	< 0.001 (*χ*^2^ = 24.4)
Hepatic decompensation at baseline, % (n)			
Encephalopathy and/or ascites	3.7 (77)	10.3 (7)	0.005 (*χ*^2^ = 7.7)
Encephalopathy	1.2 (25)	5.9 (4)	0.012
Ascites	3.1 (62)	5.9 (4)	0.151
HCC history, % (n)	3.1 (62)	5.9 (4)	0.151
F4, % (n)	45.4 (922)	76.5 (52)	< 0.001 (*χ*^2^ = 25.5)
CP, in relation to F4, % (n)	*N* = 922	*N* = 52	
B	12.4 (114)	19.2 (10)	0.148 (*χ*^2^ = 2.1)
C	0.7 (6)	0	1.0
B/C	13.0 (120)	19.2 (10)	0.2 (*χ*^2^ = 1.6)
HIV co-infection, % (n)	1.6 (32)	5.9 (4)	0.025
HBV co-infection (HBsAg+), % (n)	0.5 (11)	2.9 (2)	0.061

DAA, direct-acting antiviral; SD, standard deviation; BMI, body mass index; IFN, interferon; RBV, ribavirin; ASV, asunaprevir; DCV, daclatasvir; LDV, ledipasvir; SOF, sofosbuvir; OBV, ombitasvir; PTV, paritaprevir; r, ritonavir; DSV, dasabuvir; GZR, grazoprevir; EB, elbasvir; SMV, simeprevir; GLE, glecaprevir; PIB, pibrentasvir; VEL, velpatasvir; VOX, voxilaprevir. HCC, hepatocellular carcinoma; GT, genotype; F, fibrosis; CP, Child-Pugh scale; HIV, human immunodeficiency virus; HBV, hepatitis B virus; HBsAg, Hepatitis B virus surface antigen.

### Treatment safety

Diabetic patients were significantly more likely to experience treatment discontinuation or modification of RBV dose, so less frequently completed therapy as scheduled (95.5% vs. 97.7%, *p* < 0.001) (Table 5). The primary reasons for treatment discontinuation among diabetic patients were liver decompensation (*n* = 9), death (*n* = 8), and lack of adherence (*n* = 7).

Compared to patients without DM, they were also more likely to report AEs (23.1% vs. 16.5%, *p* < 0.001), including those classified as serious AEs (3.4% vs. 1.2%, *p* < 0.001), and those leading to discontinuation of therapy (1.1% vs. 0.5%, *p* = 0.001). Decompensation of liver function as a reason for discontinuation of therapy was significantly more frequent in patients with DM compared to those without (0.4% vs. 0.2%, *p* = 0.014). Among cirrhotic patients, ascites emerged during treatment and were more prevalent in the diabetic group (3.6% vs. 2.2%, *p* = 0.008), but rates of hepatic encephalopathy (2.1% vs. 1.4%) and gastrointestinal bleeding (0.5% vs. 0.6%) did not differ significantly across groups. Death rates during therapy and the 12-week follow-up period after treatment were also higher in the diabetic group, but none of the reported deaths were associated with the therapy.

## Discussion

This large real-world cohort study investigated clinical and metabolic differences between HCV-infected patients with and without T2DM, aiming to understand how diabetes influences the course and treatment of CHC. Among nearly 19,000 patients treated with DAAs, those with diabetes showed significantly more advanced liver disease, a distinct demographic and clinical profile (older age, male predominance, and more comorbidities), and a slightly lower treatment success rate compared to non-diabetic patients. Importantly, while SVR12 rates were lower in the diabetic group, multivariate analysis revealed that T2DM was not an independent predictor of treatment failure, suggesting that diabetes alone does not compromise DAA effectiveness in the absence of other risk factors. These findings are consistent with prior research indicating that while diabetes is associated with more severe liver disease, it does not independently reduce the likelihood of achieving SVR12 with DAA therapy.

According to the 2021 International Diabetes Federation data, 3.3% of the Polish population aged 20–79 had a confirmed DM diagnosis, though undiagnosed cases in this age group are estimated at 1.7 million, highlighting a significant underestimation of diabetes prevalence^24^. Among our cohort, 11.5% of HCV-infected patients had DM, consistent with prior studies reporting a higher DM prevalence among HCV patients^14–16^. This reinforces the association between HCV infection and increased metabolic disorder risk, attributed to viral effects on insulin resistance and metabolic regulation^25,26^.

Our analysis showed diabetic patients were significantly older (with over 83 aged ≥ 50 years), predominantly male, and more likely to have obesity. Notably, 83% of diabetic patients were aged ≥ 50 years compared to 48.6% in the non-diabetic group. These demographic patterns align with findings from Polish and global studies^27–29^. Furthermore, diabetic patients exhibited more advanced liver disease, with 48% having stage F4 fibrosis compared to 21.8% of non-diabetics. A higher proportion also had decompensated liver function at baseline. These results are consistent with evidence linking diabetes and insulin resistance to accelerated fibrosis progression^30–32^. Insulin resistance and hyperglycemia likely exacerbate fibrosis through hepatic stellate cell activation, promoting fibrogenesis^33^. Although some studies have not confirmed these links, the majority of evidence supports an association between metabolic dysfunction and fibrosis^30–32,34,35^. Furthermore, a large Taiwanese cohort study revealed a two- to threefold increased risk of cirrhosis and liver decompensation among HCV-infected individuals who developed diabetes^32^.

This interaction may also contribute to the increased risk of HCC observed in patients with diabetes. Our study highlighted a higher incidence of HCC among diabetic patients, with 3.7% reporting a history of HCC compared to 1.3% in non-diabetic. Prior research corroborates this association, showing diabetes increases HCC risk in HCV patients due to compounding metabolic and inflammatory effects^36,37^. Interestingly, this risk is not as pronounced in hepatitis B, underscoring the unique HCV-diabetes interplay^38^.

HCV genotype distribution also differed between groups. GT1b was significantly more common among diabetic patients (80.5% vs. 72.9%), while GT3 and GT4 were less prevalent. Although studies report conflicting results regarding GT-specific diabetes risk, our findings align with regional differences observed in other cohorts^16,27^. Adding further complexity, a U.S.-based study demonstrated that HCV GT2a was disproportionately observed in diabetic patients, with 29% of HCV-RNA-positive diabetic patients harboring this genotype compared to only 3% of HCV-infected controls^39^. These results suggest that the relationship between specific GTs and diabetes may vary by population, geographical region, or study design.

Treatment outcomes were slightly less favorable in diabetic patients, with SVR12 rates of 96.8% compared to 97.7% in non-diabetics (*p* = 0.013). Subgroup analysis identified male sex, GT3 infection, advanced fibrosis, and prior treatment as independent predictors of virologic failure in diabetic patients, rather than diabetes alone. These findings suggest DM amplifies existing risk factors rather than directly influencing treatment efficacy. Interestingly, patients with liver cirrhosis achieved comparable SVR12 rates regardless of the presence of DM. Our findings contrast with recent studies suggesting that DM per se does not influence the virologic response to DAAs^40^. Our multivariate analysis indicates that virologic failure in diabetic patients was independently associated with several factors, including male sex, presence of cirrhosis, GT3 infection, prior treatment history, and the use of pangenotypic regimens. In the entire population of HCV-infected patients, virologic failure was similarly influenced by male sex, concomitant medications, GT3 infection, advanced liver fibrosis (F4), more severe liver disease classified as Child-Pugh B or C, and prior treatment history. Notably, DM was not identified as an independent predictor of virologic failure in this broader analysis. Given this, we chose to include diabetes as a variable in the overall multivariate model rather than conducting separate analyses, allowing us to evaluate its role as a predictor while accounting for other influencing factors. This approach provides a comprehensive assessment of diabetes within the entire cohort rather than isolating it as an independent subgroup. Most of these predictors align with factors commonly associated with treatment failure in the general HCV population, suggesting that the presence of DM amplifies the impact of these established risk factors rather than acting as an isolated determinant^41^.

Our findings showed that patients with GT3 and coexisting T2DM had lower SVR rates compared to non-diabetic patients. This observation is consistent with existing literature suggesting that GT3 is associated with a more aggressive disease course, including higher rates of hepatic steatosis, rapid fibrosis progression, and increased risk of hepatocellular carcinoma^42^. Although T2DM alone was not an independent predictor of SVR failure in our cohort, the combination of GT3 infection and T2DM may present a higher-risk phenotype, potentially due to enhanced metabolic dysregulation and immune dysfunction. Previous studies have identified GT3 as a genotype with historically lower SVR rates in certain DAA regimens^41^. However, direct data on the interplay between GT3 and T2DM in relation to DAA outcomes remain limited, underscoring the need for further targeted research in this subgroup.

The overall safety profile of DAA therapy in our study was favorable, with high treatment tolerability and low rates of therapy discontinuation, and it was consistent with existing data on DAA therapy^43^. However, safety outcomes were less optimal in diabetic patients, who experienced adverse events, including severe ones, more frequently and had a lower treatment completion rate. Common issues such as fatigue, anemia, and pruritus were more prevalent in this group. Importantly, despite a higher mortality rate among diabetic patients, none of the fatalities were therapy-related, affirming the safety of DAAs even in this higher-risk population.

Our study aligns with existing evidence showing that T2DM contributes to accelerated liver disease progression and adverse outcomes in HCV-infected patients. Previous studies have also demonstrated that successful antiviral treatment with DAAs can lead to metabolic improvements in diabetic patients. Shengir et al. reported that achieving SVR was associated with enhanced insulin sensitivity, reductions in fasting glucose levels, and decreased risk of developing new-onset diabetes or requiring antidiabetic therapy^44^. These observations support the notion that viral eradication not only improves liver-related outcomes, but also has beneficial systemic metabolic effects, further underscoring the importance of early antiviral therapy in this high-risk population. However, it is important to note that the long-term benefits of SVR on glycemic control are still debated. For instance, Li et al. observed that although there was an initial improvement in HbA1c levels after achieving SVR, this effect did not persist over time, with levels returning to baseline after approximately 30 months^45^. This finding contrasts with studies that report lasting benefits in insulin sensitivity and glucose levels following SVR. Such discrepancies underscore the need for further research to clarify the long-term metabolic effects of SVR in patients with coexisting diabetes.

This study has some limitations. The retrospective design and reliance on real-world data introduce potential biases and underreporting of adverse events. Additionally, the impact of steatotic liver disease, which may influence liver function and outcomes, was not accounted for. Nonetheless, the study’s strengths include its large sample size, data from 22 specialized hepatology centers, and detailed patient characterization, and low rate of patients lost to follow-up, enhancing the reliability and generalizability of the findings.

## Conclusions

HCV-infected patients with comorbid DM were older, predominantly male, had higher BMI, and exhibited more advanced liver disease compared to non-diabetic patients. While DAA therapy was highly effective overall, diabetic patients experienced slightly lower treatment success rates and a worse safety profile. Importantly, DM was not an independent predictor of virologic failure. This suggests that in the absence of negative prognostic factors—such as male sex, GT3 infection, cirrhosis, or prior treatment—DM alone does not significantly reduce treatment success.

**Table 5:** Safety of DAA therapy in patients with and without diabetes.

Parameter	All patients (*n* = 18968)	Diabetic group (*n* = 2179)	Non-diabetic group (*n* = 16789)	*p*-value
Treatment course, % (n)				
According to schedule	97.4 (18476)	95.5 (2080)	97.7 (16396)	< 0.001 (*χ*^2^ = 37.5)
Therapy modification	1.3 (239)	2.3 (51)	1.1 (188)	
Therapy discontinuation	1.1 (215)	1.9 (42)	1.0 (173)	
No data	0.2 (38)	0.3 (6)	0.2 (32)	
Serious AEs, % (n)	1.4 (270)	3.4 (74)	1.2 (196)	< 0.001 (*χ*^2^ = 68.3)
AEs leading to treatment discontinuation, % (n)	0.6 (115)	1.1 (24)	0.5 (91)	0.001 (*χ*^2^ = 10.0)
Treatment discontinuation due to hepatic decompensation, % (n)	0.2 (37)	0.4 (9)	0.2 (28)	0.014 (*χ*^2^ = 6.0)
Patients with at least one AE, % (n)	17.3 (3279)	23.1 (503)	16.5 (2776)	< 0.001 (*χ*^2^ = 57.9)
Weakness/fatigue	6.8 (1287)	8.8 (191)	6.5 (1096)	< 0.001 (*χ*^2^ = 15.3)
Anemia	1.2 (234)	2.5 (54)	1.1 (180)	< 0.001 (*χ*^2^ = 31.3)
Headaches	2.4 (463)	1.7 (37)	2.5 (426)	0.017 (*χ*^2^ = 5.7)
Itchy skin	1.6 (303)	2.6 (57)	1.5 (246)	< 0.001 (*χ*^2^ = 16.2)
Death, % (n)	0.6 (115)	1.4 (31)	0.5 (84)	< 0.001 (*χ*^2^ = 27.2)

DAA, direct-acting antiviral, AEs, adverse events.

## Data Availability

The data that support the findings of this study are not openly available due to reasons of sensitivity and are available from the corresponding author upon reasonable request.
